# Associations of infant milk feed type on early postnatal growth of offspring exposed and unexposed to gestational diabetes in utero

**DOI:** 10.1007/s00394-015-1057-0

**Published:** 2015-09-28

**Authors:** Izzuddin M. Aris, Shu E. Soh, Mya Thway Tint, Seang Mei Saw, Victor S. Rajadurai, Keith M. Godfrey, Peter D. Gluckman, Fabian Yap, Yap Seng Chong, Yung Seng Lee

**Affiliations:** 1 Singapore Institute for Clinical Sciences, Agency for Science, Technology and Research, Singapore, Singapore; 2 Department of Paediatrics, Yong Loo Lin School of Medicine, National University of Singapore, 1E Kent Ridge Road, NUHS Tower Block – Level 12, Singapore, 119228 Singapore; 3 Department of Obstetrics and Gynaecology, Yong Loo Lin School of Medicine, National University of Singapore, Singapore, Singapore; 4 Saw Swee Hock School of Public Health, National University of Singapore, Singapore, Singapore; 5 Department of Neonatology, KK Women’s and Children’s Hospital, Singapore, Singapore; 6 MRC Lifecourse Epidemiology Unit and NIHR Southampton Biomedical Research Centre, University of Southampton and University Hospital Southampton NHS Foundation Trust, Southampton, UK; 7 Liggins Institute, University of Auckland, Auckland, New Zealand; 8 Department of Paediatrics, KK Women’s and Children’s Hospital, Singapore, Singapore; 9 Khoo Teck Puat-National University Children’s Medical Institute, National University Health System, Singapore, Singapore

**Keywords:** Gestational diabetes, Infant milk feeding, Offspring growth

## Abstract

**Purpose:**

Infants on prolonged breastfeeding are known to grow slower during the first year of life. It is still unclear if such effects are similar in offspring exposed to gestational diabetes (GDM) in utero. We examined the associations of infant milk feeding on postnatal growth from birth till 36 months of age in offspring exposed and unexposed to GDM.

**Methods:**

Pregnant mothers undertook 75 g 2-h oral glucose tolerance tests at 26–28 weeks of gestation for GDM diagnosis. Up to 9 measurements of offspring weight and length were collected from birth till 36 months, and interviewer-administered questionnaires were used to ascertain the duration of breastfeeding.

**Results:**

There was a statistically significant interaction between GDM status and breastmilk intake by any (*p*_interaction_ = 0.038) or exclusive/predominant breastfeeding (*p*_interaction_ = 0.035) for the outcome of conditional weight gain. In offspring of non-GDM mothers (*n* = 835), greater breastmilk intake (BF ≥ 4 milk months) was associated with lower conditional gains in weight [B (95 % CI) −0.48 (−0.58, −0.28); *p* < 0.001] within the first year of life, as well as decreasing weight SDS velocity [−0.01 (−0.02, −0.005); *p* < 0.001] and BMI SDS velocity [−0.008 (0.01, −0.002); *p* = 0.008] across age in the first 36 months. In offspring of GDM mothers (*n* = 181), however, greater breastmilk intake was associated with increased conditional gains in weight [0.72 (0.23, 1.20); *p* = 0.029] and BMI SDS [0.49 (0.04, 0.95); *p* = 0.04] in the first 6 months and did not demonstrate the decreasing weight and BMI SDS velocity observed in offspring of non-GDM mothers.

**Conclusions:**

The reduced weight gain in the first year of life conferred by greater breastmilk intake in non-GDM children was not observed in GDM children.

**Clinical trial registration:**

This study is registered under the Clinical Trials identifier NCT01174875; http://www.clinicaltrials.gov/ct2/show/NCT01174875?term=GUSTO&rank=2.

**Electronic supplementary material:**

The online version of this article (doi:10.1007/s00394-015-1057-0) contains supplementary material, which is available to authorized users.

## Introduction

The gestational and early postnatal periods have been identified as critical windows for developing risk of obesity later in life. Developmental influence on obesity risk originating from the maternal intrauterine environment has been put forth as one of the mechanisms which confer susceptibility to excessive adiposity in adulthood [1]. Recent studies have established that maternal hyperglycaemia during pregnancy is associated with increased birth size and excessive neonatal adiposity [2–4]. Prenatal exposure to increased glucose from the mother may also contribute to excessive weight gain of offspring born to diabetic mothers [5]. Infants of mothers with pre-gestational type-1 [6] or type-2 diabetes mellitus [7] have also been shown to be predisposed to develop overweight and obesity during childhood, supporting the notion that the long-term consequences of exposure to diabetes in utero on future obesity are independent of mother’s diabetes type.

In addition, infant nutrition during the early postnatal period has been identified as a critical window for later obesity risk [8]. Many studies have extensively examined the relationship between breastfeeding with growth and long-term obesity risk, highlighting that periods of long and exclusive breastfeeding is associated with slower growth in the first year of life [9, 10], and may have a protective effect on development of obesity [11–13]. The mechanisms involved, although poorly understood, has been thought to be mediated through slower growth associated with breast rather than formula feeding—the growth acceleration hypothesis [14]. Findings from other large epidemiological studies have confirmed the growth-accelerating effects of formula throughout infancy [15, 16], which may contribute to increased risk of obesity. Thus, breastfeeding has been recommended as a plausible solution to protect the offspring from the consequences of exposure to an adverse intrauterine environment, such as maternal diabetes [17, 18]. Unfortunately, our current understanding on how exposure to gestational diabetes (GDM) may influence the relationship between breastfeeding and postnatal infant growth is cluttered by the practice of combining variations of diabetes (type I, type II, GDM) into a single risk category [19, 20], despite the known differences in aetiologies and pathophysiology of these variations of diabetic disease [21]. Additionally, given the paucity of existing data on prenatal metabolic exposures and infant growth in Asian populations, and that the Asian phenotype and susceptibility towards metabolic disease differs from Europeans [22], further studies in Asian population would be merited. Thus, we sought to explore the associations of breastmilk intake on growth amongst offspring exposed and unexposed to GDM in utero and hypothesized that reduced breastmilk intake may result in accelerated adiposity gain in infants of GDM mothers.

## Methods

### Study population

This study is embedded in the Growing Up in Singapore Towards Healthy Outcomes (GUSTO) mother-offspring cohort, which has been previously described in detail [23]. Briefly, pregnant women in their first trimester were recruited from the two major public hospitals with obstetric services in Singapore, the KK Women’s and Children’s Hospital and National University Hospital from June 2009 till September 2010. Subjects approached were Singapore citizens or permanent residents who were of Chinese, Malay or Indian ethnicity with homogeneous parental ethnic background. Those who were on chemotherapy, psychotropic drugs or diabetes mellitus were excluded from the study. Of the 3751 screened, 2034 met these criteria and 1247 women (response rate 61.3 %) were recruited. Of the eligible pregnant women recruited, 1152 women had singleton naturally conceived pregnancies (Online Resource 1). This study was approved by both National Healthcare Group Domain Specific Review Board and Singhealth Centralized Institutional Review Board.

### Assessment of gestational age

Gestational age (GA) was assessed by ultrasonography. In all women, GA was first assessed in the first ultrasound dating scan during recruitment in the first trimester. Scans were conducted in a standard manner at both hospitals by trained ultrasonographers. GA was reported in completed weeks.

### Oral glucose tolerance testing

All subjects underwent a 2-h 75-g oral glucose tolerance test (OGTT) after an overnight fast between 26 and 28 weeks of gestation, and venous plasma glucose was measured by colorimetry [Advia 2400 Chemistry system (Siemens Medical Solutions Diagnostics, Deerfield, IL, USA) and Beckman LX20 Pro analyser (Beckman Coulter, USA)]. Maternal height and weight were measured during this visit. During the study period, glucose management was performed when mothers were diagnosed with GDM by World Health Organization (WHO) criteria (fasting or 2-h plasma glucose concentrations greater than 7.0 or 7.8 mmol/L, respectively). Results of the study were communicated to health practitioners, and mothers who were positively diagnosed were placed under either a diet or insulin treatment for management. Mothers with elevated fasting or 2-h plasma glucose were subjected to the same glucose management protocol. Questionnaires were administered during the visit to ascertain demographics and social economic status.

### Anthropometry measurements

Anthropometric measurements of offspring weight and length were obtained at birth, and at 3, 6, 9, 12, 15, 18, 24 and 36 months of age. Infant weight from birth till 18 months was measured to the nearest gram using a calibrated scale (SECA 334 Weighing Scale). At 24 and 36 months of age, infant weight was measured to the nearest gram using SECA 803 weighing scale. Recumbent infant length from birth to 15 months of age was measured from the top of the head to the soles of the feet using an infant mat (SECA 210 Mobile Measuring Mat) to the nearest 0.1 cm. Standing height at 18, 24 and 36 months of age was measured using the SECA 213 stadiometer, from the top of the participant’s head to his or her heels to the nearest 0.1 cm. For reliability, all measurements were taken in duplicates and averaged.

### Infant milk feeding assessment

Mothers were asked on infant milk feeding practices, based on a 24-h recall, at routine house visits when the infants were 3, 6, 9 and 12 months of age. In accordance with WHO guidelines [24], milk feeding practices were classified into exclusive, predominant and partial breastfeeding, and formula feeding. In our data collection, breastmilk intake either directly from the breast or expressed, were classified as breastfeeding. Exclusivity weights were assigned to each feeding practice using weights from 0 and 1, with exclusive breastfeeding having a weight of 1 and exclusive formula feeding having a weight of 0. Infants who were on predominant breastfeeding were given a weight of 0.75, and infants on partial breastfeeding were given a weight of 0.5. The sum of months of exclusive breastfeeding and the weighted months of predominant and partial breastfeeding [duration of exclusive breastfeeding (months) + duration of predominant breastfeeding (months) × exclusivity weight + duration of partial breastfeeding (months) × exclusivity weight] was then calculated to estimate breastmilk intake by any breastfeeding as a milk-month measure, divided into 3 categories (No BF, BF < 4 and ≥4 milk months). We also calculated the sum of months of exclusive breastfeeding and the weighted months of predominant breastfeeding [duration of exclusive breastfeeding (months) + duration of predominant breastfeeding (months) × exclusivity weight] to estimate breastmilk intake by only exclusive/predominant breastfeeding as a milk-month measure, divided into three categories (No Full BF, Full BF < 4 and ≥4 milk months).

### Statistical analysis

Descriptive statistics were reported as means and standard deviations for continuous variables and percentages for categorical variables. Age- and gender-specific standard deviation scores (SDS) were calculated for weight, length and body mass index (BMI) for infants at all time points, referencing the local Singapore population [25]. Conditional growth in weight and BMI SDS were derived from residuals resulting from regression of SDS for measurement at a specific time point on SDS for measurements at all preceding ages [26]. Multivariable linear regression analyses were used to estimate the association between estimated breastmilk intake with offspring weight and BMI conditional growth from birth to 36 months, in all cases adjusting for ethnicity, parity, maternal age, maternal education, maternal BMI at 26–28 weeks gestation and gestational age at delivery.

Additionally, we examined the longitudinal effect of estimated breastmilk intake on weight and BMI SDS trajectory using linear mixed effects (LME) models, which take into account correlation between repeated measures on the same individual, and allows for incomplete outcome data assuming that these data are missing-at-random. Maximum likelihood was the method of estimation and an unstructured working covariance matrix for random effects parameters (intercept and slope) was chosen. The Akaike information criterion statistic facilitated model selection and final models included linear, quadratic and cubic terms for children’s ages and age-milk intake interaction to estimate the change in weight and BMI SDS over time associated with estimated breastmilk intake. Besides the fixed effect of age, we allowed for a random intercept and random linear slope for age. Separate LME models were constructed for offspring of GDM and non-GDM mothers. All analysis was performed using Stata 13.0 (Statacorp, Texas).

## Results

Complete data on glucose levels were available for 1016 subjects, out of which 181 subjects (17.8 %) were diagnosed with GDM at 26–28 weeks of gestation (Online resource 1). Characteristics of the study participants are described in Table 1. There was a significant difference in distribution of GDM across all three ethnicities (*p* < 0.001). Mothers with GDM were observed to be slightly older (32.3 vs. 30.0 years; *p* < 0.001), more educated (70.2 vs. 55.5 %; *p* < 0.001), had higher BMI at 26–28 weeks of gestation (27.1 vs. 25.9 kg m^−2^; *p* < 0.001) and shorter gestational age at delivery (38.0 vs. 38.3 weeks; *p* = 0.013) compared to their non-GDM counterparts. No significant differences in birth weight, length and BMI SDS, as well as breastmilk intake, were observed for offspring of GDM and non-GDM mothers.

**Table 1:** Clinical characteristics and demographics of study subjects

Maternal characteristics	GDM (*n* = 181)		No GDM (*n* = 835)		*p* value^a^
	*n*	%/mean (SD)	*n*	%/mean (SD)	
Maternal age	181	32.3 (4.8)	835	30.0 (5.1)	**<0.001**
Maternal education					**<0.001**
<12 years	54	29.8	366	44.5	
≥12 years	127	70.2	456	55.5	
Ethnicity					**0.001**
Chinese	113	62.4	448	53.7	
Malay	28	15.5	246	29.5	
Indian	40	22.1	141	16.9	
Parity					0.101
Primiparous	68	38.0	366	44.7	
Multiparous	111	62.0	453	55.3	
Maternal BMI^b^	178	27.1 (4.3)	815	25.9 (4.5)	**<0.001**
Gestational age at delivery	181	38.0 (1.7)	824	38.3 (1.4)	**0.013**
*Infant characteristics*					
Birth weight SDS^b^	181	−0.50 (1.17)	824	−0.48 (0.95)	0.886
Birth length SDS^b^	181	−0.39 (1.37)	821	−0.52 (1.14)	0.229
Birth BMI SDS^b^	181	−0.47 (1.20)	821	−0.31 (1.08)	0.090
Breastmilk intake (by any breastfeeding)					0.352
No BF	49	29.3	253	33.1	
<4 milk months	58	34.7	281	36.8	
≥4 milk months	60	35.9	230	30.1	
Breastmilk intake (by exclusive/predominant breastfeeding only)					0.061
No Full BF	99	59.3	511	66.9	
Full BF <4 milk months	26	15.6	120	15.7	
Full BF ≥4 milk months	42	25.1	133	17.4	

The bold values represent staistically significant *p* values (i.e. *p* < 0.05)

^a^ *p* value by Chi-square analysis (categorical) or two-sample *t* test (continuous)

^b^ *BMI* body mass index, *SDS* standard deviation score

**Fig. 1:**
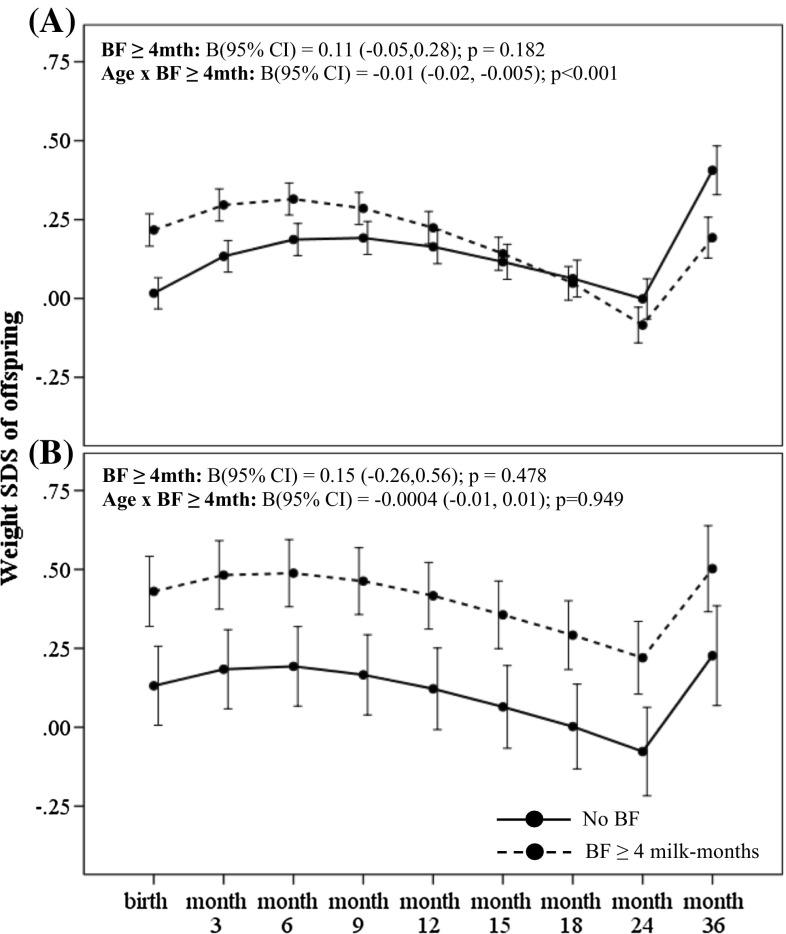
Weight SDS trajectory in the first 36 months according to breastmilk intake by any breastfeeding for offspring of **a** non-GDM and **b** GDM mothers. Adjusted for ethnicity, parity, maternal age, maternal education, maternal BMI at 26–28 weeks gestation and gestational age at delivery. Age-milk intake interaction term (i.e. Age × BF) represents the estimated change in weight and BMI SDS over time (i.e. weight and BMI SDS velocity, respectively) associated with breastmilk intake. *Solid line* No BF; *Dashed line* BF ≥ 4 months

**Fig. 2:**
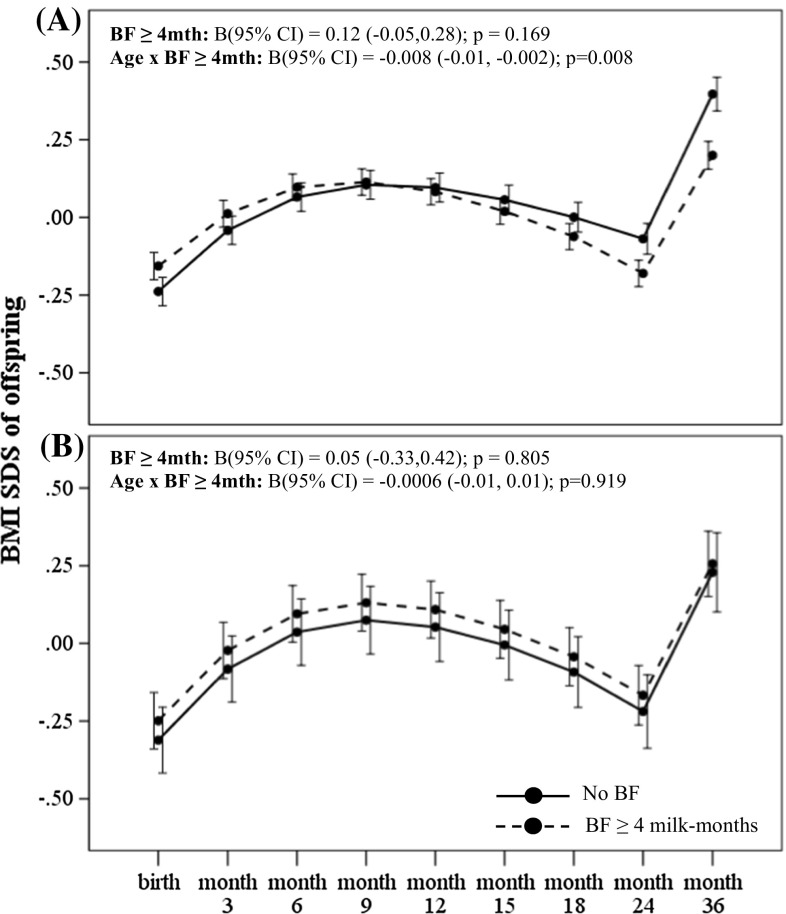
BMI SDS trajectory in the first 36 months according to breastmilk intake by any breastfeeding for offspring of **a** non-GDM and **b** GDM mothers. Adjusted for ethnicity, parity, maternal age, maternal education, maternal BMI at 26–28 weeks gestation and gestational age at delivery. Age-milk intake interaction term (i.e. Age × BF) represents the estimated change in weight and BMI SDS over time (i.e. weight and BMI SDS velocity, respectively) associated with breastmilk intake. *Solid line* No BF; *Dashed line* BF ≥ 4 months

**Fig. 3:**
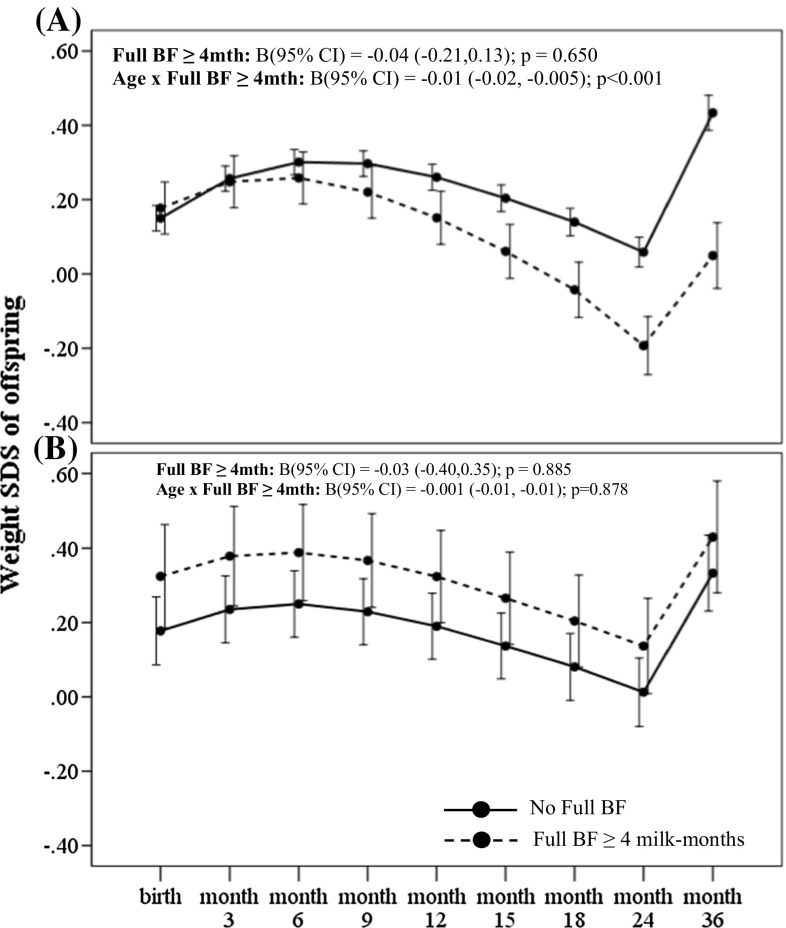
Weight SDS trajectory in the first 36 months according to breastmilk intake by exclusive/predominant breastfeeding for offspring of **a** non-GDM and **b** GDM mothers. Adjusted for ethnicity, parity, maternal age, maternal education, maternal BMI at 26–28 weeks gestation and gestational age at delivery. Age-milk intake interaction term (i.e. Age × Full BF) represents the estimated change in weight and BMI SDS over time (i.e. weight and BMI SDS velocity, respectively) associated with breastmilk intake. *Solid line* No Full BF; *Dashed line* Full BF ≥ 4 months

There was a statistically significant interaction between GDM status with breastmilk intake by any breastfeeding (F-statistic = 3.294, *p*_interaction_ = 0.038) or exclusive/predominant breastfeeding (F-statistic = 3.376, *p*_interaction_ = 0.035) for the outcome of conditional weight gain. Amongst offspring of non-GDM mothers, greater breastmilk intake by any breastfeeding (BF ≥ 4 milk months) was associated with significantly decelerated conditional gain in weight [B (95 % CI) −0.48 (−0.58, −0.28); *p* < 0.001] within the first 12 months, compared to those with no breastfeeding (Table 2). Greater breastmilk intake by exclusive/predominant breastfeeding (Full BF ≥ 4 milk months) was also associated with significantly decelerated conditional gain in weight SDS [−0.50 (−0.70, −0.30); *p* < 0.001] within the first 12 months, compared to those who were not on exclusive/predominant breastfeeding (Table 2). In a fully adjusted LME model for offspring of non-GDM mothers, greater breastmilk intake by any breastfeeding (BF ≥ 4 milk months) was associated with decreased weight and BMI SDS velocity across age in the first 36 months compared to those with no breastfeeding, with an estimated rate of decrease in 0.01 SDS units per month (95 % CI −0.02, −0.005; *p* < 0.001) for weight (Fig. 1a) and 0.008 SDS units per month (95 % CI −0.01, −0.002; *p* = 0.008) for BMI (Fig. 2a). Similarly, greater breastmilk intake by exclusive/predominant breastfeeding (Full BF ≥ 4 milk months) was also associated with decreased weight SDS velocity across age in the first 36 months, with an estimated rate of decrease in 0.01 SDS units per month (95 % CI −0.02, −0.005; *p* < 0.001) (Fig. 3a).

**Table 2:** Associations of estimated breastmilk intake by any breastfeeding and by exclusive/predominant breastfeeding on conditional growth of offspring in the first 36 months of life for offspring exposed and unexposed to gestational diabetes in utero

	Conditional SDS gain^#^ B (95 % CI)									
	Unexposed to GDM					Exposed to GDM				
	0–6 months	6–12 months	12–18 months	18–24 months	24–36 months	0–6 months	6–12 months	12–18 months	18–24 months	24–36 months
*Weight SDS*										
No BF	Ref					Ref				
BF < 4 months	0.12 (−0.07,0.31)	−0.12 (−0.32,0.07)	0.17 (−0.04, 0.38)	0.09 (−0.14,0.31)	−0.25 (−0.47,−0.02)	0.45 (−0.02,0.92)	−0.003 (−0.45,0.44)	−0.14 (−0.68,0.39)	0.08 (−0.41,0.58)	−0.19 (−0.67,0.28)
BF ≥ 4 months	−0.04 (−0.24,0.16)	−0.48** (**−0.68,−0.28)**	0.06 (−0.16,0.28)	0.12 (−0.12,0.35)	−0.15 (−0.38,0.08)	0.72** (**0.23,1.20)**	−0.55 (−0.99,0.07)	−0.13 (−0.68,0.42)	0.36 (−0.14,0.87)	−0.06 (−0.44,0.55)
No Full BF	Ref					Ref				
Full BF < 4 months	−0.16 (−0.39,0.06)	−0.10 (−0.33,0.13)	−0.21 (−0.04,0.46)	0.15 (−0.12,0.42)	0.11 (−0.16,0.38)	0.24 (−0.28,0.76)	−0.57 (−1.05, 0.09)	0.06 (−0.51,0.62)	0.05 (−0.48,0.57)	0.32 (−0.19,0.82)
Full BF ≥ 4 months	−0.11 (−0.31,0.09)	−0.50** (**−0.70,−0.30)**	−0.11 (−0.33,0.11)	0.17 (−0.06,0.40)	−0.03 (−0.26,0.20)	0.53* (**0.08,0.97)**	−0.55 (−0.95, 0.16)	−0.04 (−0.53,0.44)	0.20 (−0.25,0.65)	0.19 (−0.26,0.63)
*BMI SDS*										
No BF	Ref					Ref				
BF < 4 months	0.11 (−0.08,0.30)	0.009 (−0.19,0.21)	−0.004 (−0.22,0.21)	0.23 (−0.03,0.48)	−0.21 (−0.44,0.02)	0.18 (−0.26,0.62)	−0.22 (−0.71,0.28)	−0.13 (−0.73,0.46)	−0.04 (−0.59,0.52)	−0.30 (−0.85,0.25)
BF ≥ 4 months	0.17 (−0.03,0.37)	−0.18 (−0.38,0.03)	−0.27 (−0.49,−0.04)	0.14 (−0.12,0.39)	−0.20 (−0.43,0.04)	0.49* (**0.04,0.95)**	−0.36 (−0.87,0.16)	−0.29 (−0.90,0.32)	0.16 (−0.40,0.72)	−0.06 (−0.52,0.64)
No Full BF	Ref					Ref				
Full BF < 4 months	−0.17 (−0.39,0.05)	−0.04 (−0.28,0.20)	−0.09 (−0.35,0.17)	−0.09 (−0.40,0.22)	0.07 (−0.21,0.36)	0.22 (−0.27,0.71)	−0.38 (−0.90,0.16)	−0.03 (−0.68,0.62)	−0.41 (−1.03,0.22)	0.48 (−0.11,1.08)
Full BF ≥ 4 months	0.18 (−0.02,0.38)	−0.19 (−0.40,0.02)	−0.30 (−0.52,−0.08)	0.07 (−0.19,0.32)	−0.05 (−0.29,0.18)	0.58* (**0.16,0.99)**	−0.42 (−0.85,0.02)	−0.40 (−0.95,0.14)	0.08 (−0.42,0.58)	0.37 (−0.15,0.88)

The bold values represent staistically significant *p* values (i.e. *p* < 0.05)

* p < 0.05; ** *p* < 0.01

^#^Adjusted for maternal age, ethnicity, maternal education, parity, maternal BMI at 26–28 weeks gestation, gestational age at delivery

**Fig. 4:**
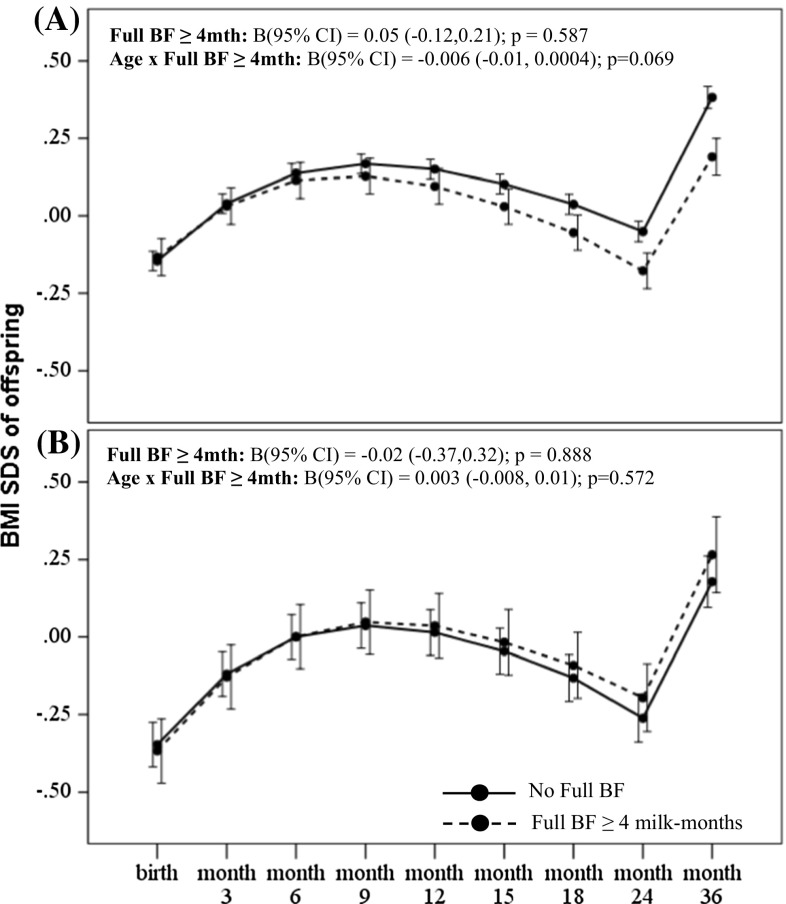
BMI SDS trajectory in the first 36 months according to breastmilk intake by exclusive/predominant breastfeeding for offspring of **a** non-GDM and **b** GDM mothers. Adjusted for ethnicity, parity, maternal age, maternal education, maternal BMI at 26–28 weeks gestation and gestational age at delivery. Age-milk intake interaction term (i.e. Age × BF) represents the estimated change in weight and BMI SDS over time (i.e. weight and BMI SDS velocity, respectively) associated with breastmilk intake. *Solid line* No Full BF; *Dashed line* Full BF ≥ 4 months

Amongst offspring of GDM mothers however, greater breastmilk intake by any breastfeeding was associated with significantly accelerated conditional gain in weight [B (95 % CI) 0.72 (0.23, 1.20); *p* = 0.004] and BMI SDS [0.49 (0.04, 0.95); *p* = 0.035] in the first 6 months compared to those with no breastfeeding (Table 2). Greater breastmilk intake by exclusive/predominant breastfeeding was also associated with significantly accelerated conditional gain in weight SDS [0.53 (0.08, 0.97); *p* = 0.021] and BMI SDS [0.58 (0.16, 0.99); *p* = 0.007] in the first 6 months, compared to those who were not on exclusive/predominant breastfeeding (Table 2). However, no significant associations with growth outcomes during the first 36 months were observed for those with breastmilk intake by exclusive/predominant breastfeeding less than 4 months. In a fully adjusted LME model for offspring of GDM mothers, there were no significant associations between breastmilk intake with weight (Figs. 1b, 3b) and BMI SDS velocity (Figs. 2b, 4b) in the first 36 months.

Additionally, we tested whether maternal BMI status (divided into normal, overweight and obese) and degree of hyperglycaemia (divided into quartiles) in GDM mothers would influence the association between breastmilk intake and offspring growth. We noted there were no statistically significant interactions between maternal BMI status with breastmilk intake (F-statistic = 0.580, *p*_interaction_ = 0.678), and between degree of hyperglycaemia with breastmilk intake (F-statistic = 0.647, *p*_interaction_ = 0.692) for the outcome of conditional weight gain in offspring of GDM mothers, suggesting that the observed association between breastmilk intake and growth in offspring GDM mothers are not mediated by both the degree of maternal hyperglycaemia and BMI.

## Discussion

In this prospective Asian birth cohort study, we have demonstrated varied associations of breastmilk intake on early postnatal growth in offspring exposed and unexposed to GDM in utero. We noted that offspring of mothers without GDM with greater breastmilk intake (i.e. ≥4 months) were associated with decelerated growth compared to those who with no breastfeeding. However, the reduced weight gain in the first year conferred by greater breastmilk intake in non-GDM children was not observed in GDM children.

Our findings on observed slower growth from greater breastmilk intake amongst non-GDM mothers are a well-described phenomenon and have been widely documented in literature. Griffiths et al. [9] similarly reported that infants of mothers without GDM who did not receive breastmilk grew faster than those whose mothers initiated breastfeeding, as did those who breastfed for 4 months or longer. A cohort study of randomly selected healthy newborns in Denmark and Iceland showed that exclusive breastfeeding beyond 2 months of age was related to lower weight gain from 2 to 6 months as well as from 6 to 12 months [10]. A recent study on the Gemini cohort of 4680 infants also showed that infants breastfed for longer periods (>4 months) was independently associated with lower growth velocity by 6.8 % [27]. Taken together with our study findings, it supports the notion that periods of long and exclusive breastfeeding may have a protective effect on development of obesity later in life. Not all studies, however, have reported such similar results. A cluster randomized trial of a breastfeeding promotion intervention modelled on the WHO/UNICEF Baby-Friendly Hospital Initiative showed that prolonged and exclusive breastfeeding accelerated weight and length gain of the infants in the first few months with no detectable deficit by 12 months old [28].

Early animal studies have suggested that milk derived from mothers with GDM may impart metabolic consequences to their offspring. It has been reported that control offspring who were fed milk from dams with GDM showed complex “malprogramming” of hypothalamic neural circuits that are critically involved in the regulation of food intake, body weight, and metabolism [29]. Longitudinal studies in humans also echoed similar observations, where breastmilk feeds from diabetic mothers during the first week of life was associated with greater relative weight and risk of overweight at 2-years, compared to offspring of diabetic mothers who were fed banked donor breastmilk [17, 18]. Other studies, however, such as that by Crume et al. [19], have reported contrasting findings where it was shown that adequate breastfeeding (≥6 breastmilk months) reduces overall body size and BMI growth velocity in the first 9 months of life amongst offspring of diabetic pregnancies. Another study also reported that breastfeeding conferred similar protective effects against overweight at 9–14 years of age in offspring of both non-diabetic and diabetic women [20]. It is important to note, however, that these studies classified type-I diabetes and GDM into a single category, which may have explained the inconsistency in findings with our study due to the differences in aetiologies of both diabetic sub-types. Our findings thus provide critical insights into this area of research, given the lack of understanding of the biochemical impact of breastmilk from GDM mothers on infant growth.

The plausible mechanisms underlying the varied effects of breastmilk intake on early postnatal growth in offspring exposed and unexposed to GDM in utero are likely multiple. In GDM mothers, researchers have postulated that differences in breastmilk constituents of diabetic and non-diabetic mothers, such as increased glucose or insulin concentrations in breastmilk of diabetic mothers may contribute to increased growth rates during early infancy [30, 31]. Concentration of ghrelin in breastmilk of GDM-lactating women has also been reported to be lower when compared to non-diabetic control samples [32]. However, there is positive significant correlation between levels of active ghrelin in 4th month breastmilk and weight gain [33]; hence, the significance of this reduced ghrelin in breastmilk of GDM mothers in our observation is uncertain. Milk from diabetic mothers may also contain more inflammatory cytokines (e.g. TNF-α, IL-6) which mimic signalling pathways characteristic of dysfunctional adipocytes of metabolic syndrome [34]. Moreover, Kjos et al. [35] had earlier demonstrated that abnormal glucose metabolism still persists postpartum amongst women with GDM, further suggesting that continued exposure to altered fuels through breastmilk may bring about consequences to offspring growth. Plagemann et al. [36] had also proposed that milk originating from diabetic mothers may have an early obesogenic effect on infant weight gain that decreases with time. Taken together, these studies suggest that differences in breastmilk composition may explain our observations of increased early growth with greater breastmilk intake in offspring of GDM mothers. Despite this, it is important to note that there may still be positive effects of breastfeeding on reducing later adiposity in offspring of diabetic mothers, as highlighted by Crume et al. [19] and Mayer-Davis et al. [20]. It has been suggested that the benefits of breastfeeding in offspring of diabetic mothers may only be observed if breastfeeding is continued beyond a certain period where breastmilk composition would have normalized over time [36]. The Kaulsdorf Cohort Study in Germany had highlighted that intake of donor breastmilk from metabolically healthy mothers prevented increased early weight gain in offspring of diabetic mothers [37], further indicating that breastmilk of a normal composition, which would result from good metabolic control of women with diabetes [38], may be beneficial to prevent future obesity risk in offspring of diabetic women. This begets the question whether proactive intervention to achieve better glycemic control in the early postpartum period of GDM mothers would help to accelerate the normalization of the breastmilk and reduce the obesogenic effect, which poses an interesting hypothesis that should be examined properly in an interventional trial.

Strengths of our study include the prospective design with high follow-up rate, along with the study of Asian ethnic groups. Given the paucity of existing data on prenatal metabolic exposures and infant growth in Asian populations, our study thus provides useful and informative data on this relationship. This study, however, is with limitations. Due to the observational nature of our study, we cannot fully rule out the possibility that residual confounding by parental attributes or family environment may affect the observed associations, as breastfeeding is a behaviour that is self-selected and women are usually not randomized to breastfeed. As with most studies on breastfeeding and infant growth, it is largely observational and hence subject to potential confounding. Socio-economic status presents as an important confounder, as mothers who are more educated tend to be more “nutrition-conscious”, more likely to breastfeed and less likely to feed poor quality diets postweaning [39, 40]. Other important potential confounding factors include maternal BMI, which is generally associated with shorter durations of breastfeeding [41], as well as maternal age, which is generally associated with greater exclusive breastfeeding [42], all of which presents a potential bias. The associations observed in our study findings have been controlled for and are independent of these potential confounders.

In conclusion, our study findings have demonstrated varied associations of breastmilk intake on early postnatal growth in offspring exposed and unexposed to GDM in utero. Whilst offspring of non-GDM mothers exhibited reduced weight gain in the first year of life conferred by greater breastmilk intake, the same effect was not observed in GDM children. It remains to be seen if our observed associations of breastmilk intake on accelerated growth amongst offspring exposed to GDM in utero have independent long-term effects in adolescence and adulthood in our cohort.

## Electronic supplementary material

Below is the link to the electronic supplementary material.

Supplementary material 1 (PDF 9 kb)
