# Supplementary figures and images for: Associations of infant milk feed type on early postnatal growth of offspring exposed and unexposed to gestational diabetes in utero
Source: Eur J Nutr. 2015 Sep 28;56(1):55–64. doi: 10.1007/s00394-015-1057-0 (PMC5290064; doi:10.1007/s00394-015-1057-0)

**Online Resource 1:** Flowchart for GUSTO Study recruitment and eventual study sample

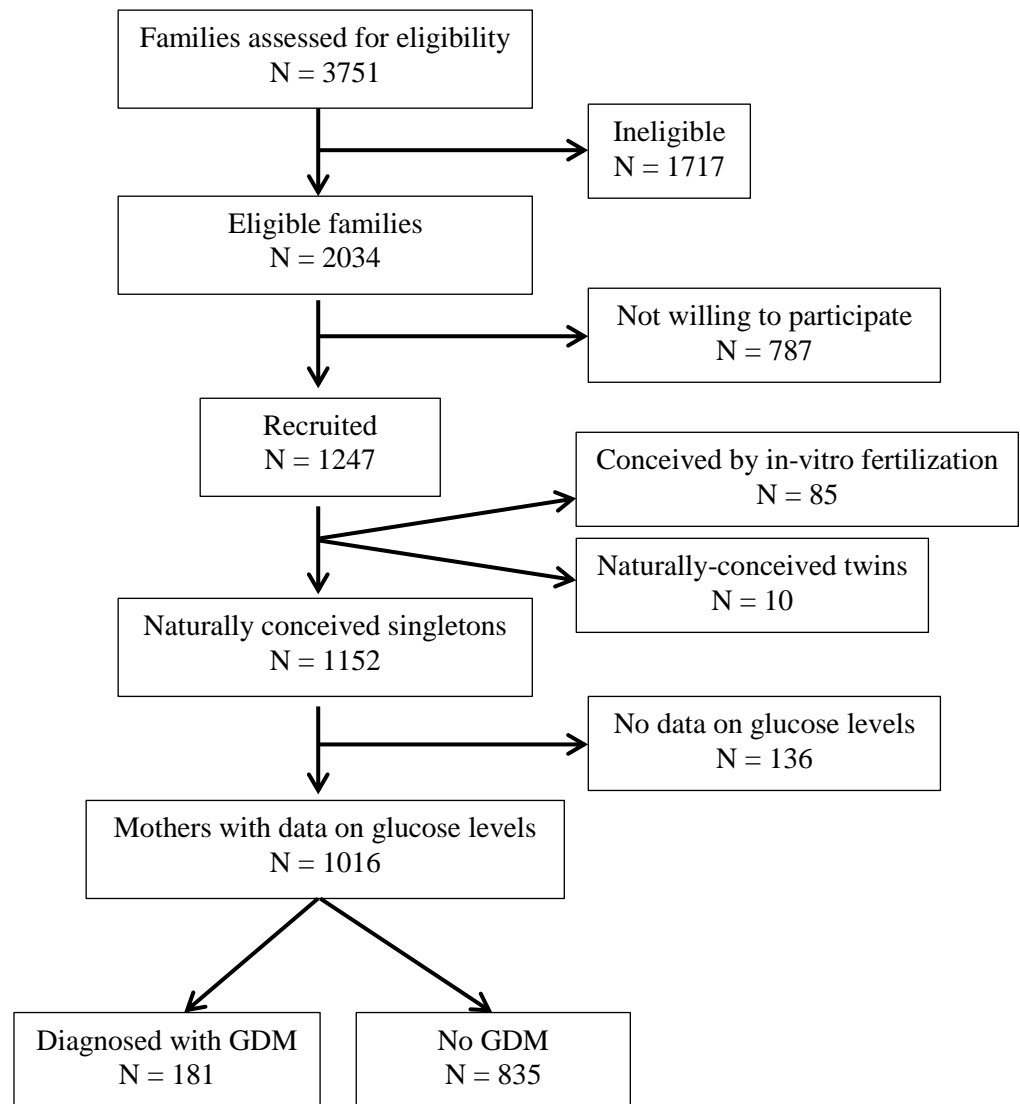

Supplement: Supplementary file 1 — Supplementary material 1 (PDF 9 kb) [file 394_2015_1057_MOESM1_ESM.pdf]
